# Binding of estrogen receptor with estrogen conjugated to bovine serum albumin (BSA)

**DOI:** 10.1186/1478-1336-2-5

**Published:** 2004-08-19

**Authors:** Yasuto Taguchi, Mirek Koslowski, Donald L Bodenner

**Affiliations:** 1 Obstetrics and Gynecology, University of Arkansas for Medical Sciences, 4301 West Markham Street, Little Rock, AR 72205, USA; 2 Endocrinology and Metabolism, Emory University School of Medicine and Veterans Affairs Medical Center, Atlanta, Georgia 30033, USA; 3 Geriatrics, University of Arkansas for Medical Sciences, 4301 West Markham Street, Little Rock, AR 72205, USA

## Abstract

**Background:**

The classic model of estrogen action requires that the estrogen receptor (ER) activates gene expression by binding directly or indirectly to DNA. Recent studies, however, strongly suggest that ER can act through nongenomic signal transduction pathways and may be mediated by a membrane bound form of the ER. Estradiol covalently linked to membrane impermeable BSA (E_2_-BSA) has been widely used as an agent to study these novel membrane-associated ER events. However, a recent report suggests that E_2_-BSA does not compete for E_2 _binding to purified ER *in vitro*. To resolve this apparent discrepancy, we performed competition studies examining the binding of E_2 _and E_2_-BSA to both purified ER preparations and ER within intact cells. To eliminate potential artifacts due to contamination of commercially available E_2_-BSA preparations with unconjugated E_2 _(usually between 3–5%), the latter was carefully removed by ultrafiltration.

**Results:**

As previously reported, a 10-to 1000-fold molar excess of E_2_-BSA was unable to compete with ^3^H-E_2 _binding to ER when added simultaneously. However, when ER was pre-incubated with the same concentrations of E_2_-BSA, the binding of ^3^H-E_2 _was significantly reduced. E_2_-BSA binding to a putative membrane-associated ER was directly visualized using fluorescein labeled E_2_-BSA (E_2_-BSA-FITC). Staining was restricted to the cell membrane when E_2_-BSA-FITC was incubated with stable transfectants of the murine ERα within ER-negative HeLa cells and with MC7 cells that endogenously produce ERα. This staining appeared highly specific since it was competed by pre-incubation with E_2 _in a dose dependent manner and with the competitor ICI-182,780.

**Conclusions:**

These results demonstrate that E_2_-BSA does bind to purified ER *in vitro *and to ER in intact cells. It seems likely that the size and structure of E_2_-BSA requires more energy for it to bind to the ER and consequently binds more slowly than E_2_. More importantly, these findings demonstrate that in intact cells that express ER, E_2_-BSA binding is localized to the cell membrane, strongly suggesting a membrane bound form of the ER.

## Background

For many years, estrogen actions have been presumed to be mediated almost exclusively through the regulation of target gene transcription by a chromosomal bound estrogen receptor. These genomic estrogen effects are the well described interactions between the estrogen receptor and adapter transcription factors that result in activation or inhibition of the basal transcription protein machinery. However, there is a growing body of evidence that several rapid estrogen effects are non-transcriptional in nature. These rapid estrogen effects include changes of calcium flux in several cell types [1-3], MAPK activation [4,5], cAMP levels [6,7], and nitric oxide release [8]. That many of these effects are mediated by a membrane-localized estrogen receptor has been postulated for some time [9,10], but the majority of evidence supporting this hypothesis is indirect, relying on the induction of these non-genomic effects using estrogen covalently conjugated to BSA by a 6 atom hydrocarbon tether (E_2_-BSA) [11,12]. However, the relative binding efficiency of these conjugates is low and concern has been raised regarding the use of these conjugates as direct surrogates for estrogen [13]. A recent report added to this controversy by showing that commercially available E_2_-BSA is contaminated by unconjugated free E_2 _and a series of binding experiments demonstrated that E_2_-BSA was unable to bind to ER after the contaminant E_2 _was removed. [14]. These findings contradict studies where fluorescein-labeled E_2_-BSA (E_2_-BSA-FITC) specifically bound to a putative ER on the cell membrane [15-17].

Elucidation of novel membrane-associated ER effects is crucial to our understanding of the non-genomic signaling pathways of ER and other hormone receptors. Hormone-conjugated BSA is an important tool in this pursuit. We believe the contradictory results are explained by differences in the rates of binding of the bulky E_2_-BSA and E_2 _with the ER. We show that pre-incubation of E_2_-BSA with ERα results in a highly significant decrease in the binding of ^3^H-E_2_. The binding of ^3^H-E_2 _with ERα is unaffected by the simultaneous addition of E_2_-BSA. We also demonstrate that fluorescein conjugated E_2_-BSA binds to the membrane of cells that endogenously produce ERα and to HeLa cell lines stably expressing mERα.

## Results

### E_2_-BSA binding to purified estrogen receptor

**Figure 1:**
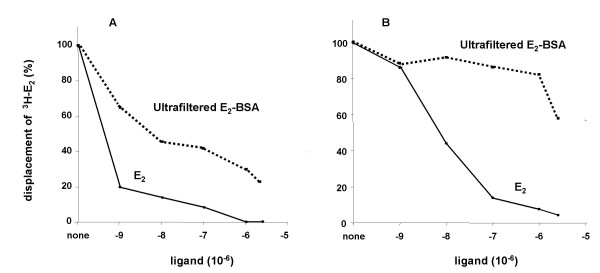
**Pre-incubation of purified hERα with E_2_-BSA competes for estradiol binding. **Purified ERα was incubated with E_2 _(solid line) or E_2_-BSA (dotted line) for four hours before (a) or concurrently with (b) the addition of labeled E_2_. Incubation was continued for another 2 h at room temperature, and at the end of this period, specific binding was determined by adsorption, removal, and counting of free labeled E_2_.

Although E_2 _is covalently attached to BSA using a relatively long six atom hydrocarbon tether, the bulky BSA moiety of E_2_-BSA still may be interfering with the binding between the estrogen molecule and the estrogen receptor. This would result in an increase in the energy of activation required for E_2_-BSA binding. If so, increasing the reaction time would allow for the establishment of an equilibrium between bound and free forms of E_2_-BSA, maximizing the amount of E_2_-BSA bound to the receptor. To test this hypothesis, E_2_-BSA free of contaminant E_2 _was prepared by ultrafiltration. Competition between the purified E_2_-BSA and labeled E_2 _for binding to purified ERα was determined after E_2_-BSA was pre-incubated with ERα and also when added at the same time as labeled E_2_. As shown in figure 1, concurrent addition of labeled E_2 _and E_2_-BSA had no effect on labeled E_2 _binding. However, a four-hour pre-incubation of E_2_-BSA with ER significantly decreased E_2 _binding. These results suggest that the large BSA molecule retards, but does not prevent binding of E_2_-BSA.

### E_2_-BSA binding to ER in intact cells

**Figure 2:**
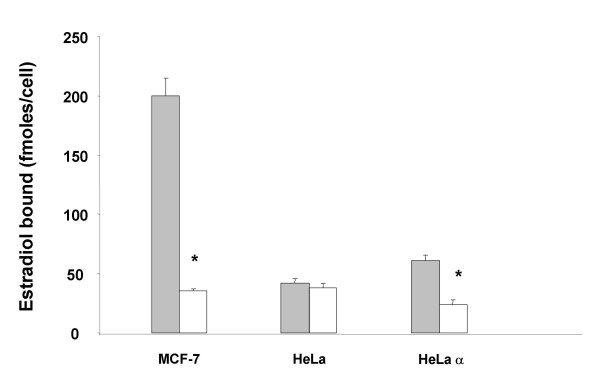
**Whole cell binding of estradiol to MC7 cells and HeLa cells stably transfected with ERα. **2 × 10^6 ^cells were incubated with ^3^H-17β-estradiol (10^-8 ^M) in the absence (solid) and presence (white) of 100 fold excess of unlabeled estradiol for 15 minutes at room temperature, washed, and placed on ice for 30 minutes. Cells were then pelleted, lysed and counted. Results are expressed as the mean +/- the SEM of 3 experiments (* p < 0.01)

**Figure 3:**
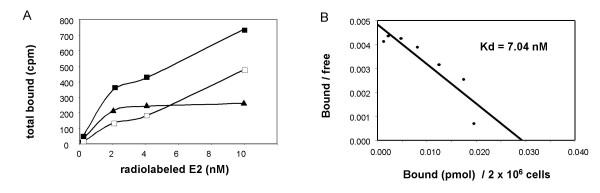
**Estradiol binding to HeLa cells stably transfected with ERα. **Subconfluent HeLa-ERα cells were trypsinized and aliquots (2 × 10^6 ^cells) incubated with several concentrations of ^3^H-17β-estradiol in the presence and absence of a 200-fold excess of cold 17β-estradiol for 30 min at 37°. Cells were then incubated on ice for 15 min, washed three times with 2 ml of ice cold 0.2% BSA-saline and pelleted by centrifugation at 1,5000 rpm for 10 min at 4°C. Cells were lysed by the addition of 100 ul of lysis buffer, vortexed and counted. a) Representative binding results of 3 independent experiments with total binding (solid box), non-specific binding (open box), and specific binding (triangle). b) Scatchard analysis of binding results.

**Figure 4:**
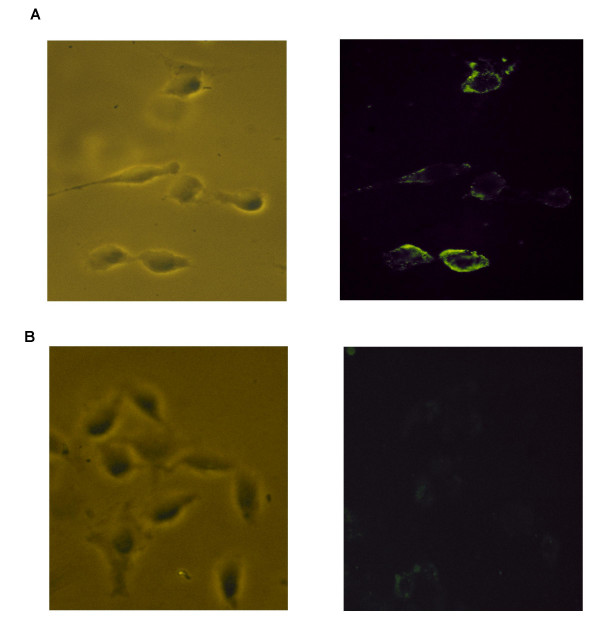
**Membrane localization of ERα. **HeLa cells stably transfected with ERα(a) and native HeLa cells (b) were incubated with fluorescein labeled membrane impermeable BSA conjugated to estradiol (E_2_-BSA-FITC) and visualized under phase contrast bright field and with UV light with an excitation filter for FITC.

Non-genomic actions of the estrogen receptor are now well established. Several investigators have demonstrated that fluorescein labeled E_2_-BSA (E_2_-BSA-FITC) binds to the cell membrane, suggesting that a form of the estrogen receptor is present within the cell membrane and capable of binding to extracellular E_2_. Specific binding of E_2_-BSA-FITC to this membrane-localized form of the ER would further establish that E_2 _conjugated to the BSA molecule is capable of binding to the ER. To examine this possibility, E_2_-BSA-FITC binding studies were performed with MC7 cells that contain endogenous ERα and with ER-deficient HeLa cells stably transfected with the ERα (HeLa-ERα). Expression of ERα within the HeLa cells was established by demonstrating specific binding of labeled E_2 _to HeLa-ERα, but not native HeLa cells (figure 2). Scatchard analysis of the binding of E_2 _to HeLa-ERα cells showed that although weakly expressed, the Kd for the expressed ERα was 7.04 nM, similar to published values (figure 3). HeLa-ERα cells, but not native HeLa cells, exhibited fluorescent staining of the cell membrane after incubation with E_2_-BSA-FITC (figure 4). The heterogeneous staining pattern reflected the low level of ERα expression. This fluorescence was not seen when HeLa-ERα cells were incubated with BSA conjugated to fluorescein alone (data not shown).

**Figure 5:**
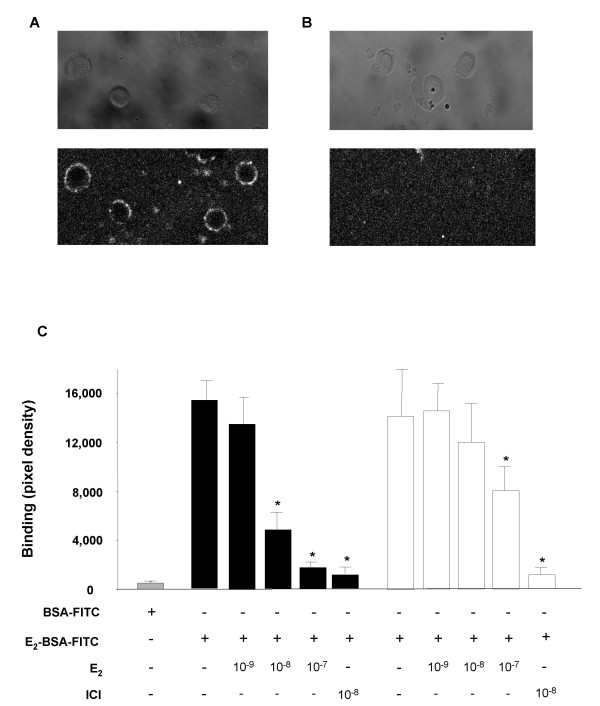
**E_2 _competition with E_2_-BSA-FITC binding. **MC7 cells (black bars) or HeLa-ERα (white bars) were incubated with vehicle, various concentrations of E_2_, or ICI 182,780 (10^-8 ^M) for 30 minutes and then incubated an additional 30 minutes with E_2_-BSA-FITC or BSA-FITC alone (grey bar). Cells were fixed, and visualized by confocal microscopy. Digitized images were inverted to black on white and pixel density for each cell was determined by averaging the density across the cell membrane at four orthogonal points. Each bar represents >20 cells counted +/- SEM. (* p < 0.05).

To establish the specificity of E_2_-BSA binding, MC7 cells and HeLa-ERα cells were incubated with E_2_-BSA-FITC after pre-incubation with various concentrations of E_2 _and the anti-estrogen ICI-182,780. As shown in figure 5, fluorescence was lost in both cell types in a dose dependent manner with increasing concentrations of E_2_. Fluorescence was almost completely eliminated by pre-incubation with the specific competitor ICI-182,780. BSA conjugated to FITC alone did not bind. These results suggest that estrogen covalently bound to BSA can bind to ER in a biologically significant manner.

**Figure 6:**
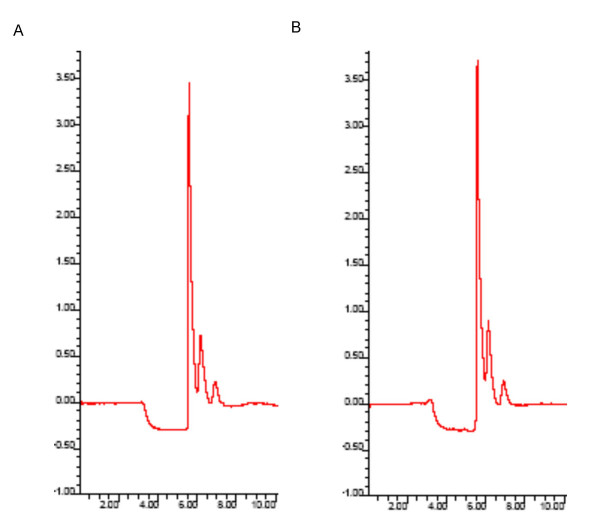
**Stability of E_2_-BSA-FITC. **E_2_-BSA-FITC (10^-8 ^M in estrogen) was placed in empty wells or wells containing MC7 cells and incubated for 30 minutes at 4°C. E_2_-BSA-FITC was detected by reverse phase HPLC using a methanol-water gradient from 80% methanol to 50% methanol over 30 minutes at 1.0 ml/min. The assay was run in triplicate. Representative spectra are shown for E_2_-BSA-FITC alone (A) and E_2_-BSA-FITC incubated with MC7 cells (B).

The possibility that E_2_-BSA-FITC could be degraded during incubation with intact cells was examined using HPLC. E_2_-BSA-FITC was incubated in empty wells or wells containing MC7 cells under the same conditions employed for the binding studies described above. Media was removed from the cells and HPLC performed with a reverse phase column. Peaks were visualized using a scanning fluorescence detector. Aqueous solutions of E_2_-BSA-FITC produced a single peak with a retention time of 5.5 minutes using a methanol-water gradient from 80% to 50% over 30 minutes at 1 ml/minute. E_2 _and E_2_-BSA did not fluoresce at the excitation and emission wavelengths used (data not shown). Spectra obtained from media containing E_2_-BSA-FITC alone and media containing E_2_-BSA-FITC incubated with MC7 cells are shown in figure 6. The average area under the curve for E_2_-BSA-FITC was the same (p < 0.05) for solutions incubated in the presence (44,556 +/- 432) and absence (43,436 +/- 289) of MC7 cells (p, 0.05). These results demonstrate that E_2_-BSA-FITC is stable under the culture conditions employed for the binding experiments.

## Discussion

The cellular effects elicited by estrogen [11,12,18-20,3] testosterone [3,21,22] and progesterone [23-25] covalently conjugated to membrane impermeable BSA have been attributed to non-genomic actions mediated by membrane associated hormone receptors. The use of these reagents for this purpose remains controversial for several reasons. A recent report demonstrated that E_2_-BSA does not bind to purified ER in competition assays with labeled E_2 _[14]. The studies were performed when E_2_-BSA or cold E_2 _were added concurrently with labeled E_2_. We obtained similar results under these conditions. However, pre-incubation of E_2_-BSA with purified ER results in significant competition with labeled E_2_. These conflicting results may be explained by differences in the rate of binding between E_2 _and E_2_-BSA. E_2_-BSA is a large, bulky molecule similar in size to the ER and is probably spherical in general structure as is the parent BSA molecule. The BSA protein conformation immediately adjacent to the covalently bound estrogen undoubtedly provides substantial steric hindrance to the proper presentation of conjugated E_2 _to the binding pocket of ER. The increased size of the E_2_-BSA molecule would also reduce the rate of its diffusion compared with the smaller E_2_. Correct orientation of E2 in the ER binding pocket is also impeded by the restraint on three-dimensional movement imposed by the six atom spacer used to connect BSA and E_2_. Lastly, the use of E_2_-BSA solutions that are formulated in terms of the molarity of total bound-E_2 _probably overestimates the amount of E_2 _available for binding. The rate of binding between E_2 _and ER can be expressed using the second order rate equation: rate = k [E_2_] [ER], where [E_2_] is the concentration of estradiol, [ER] is the concentration of the ER, and k the rate constant.

Commercially available E_2_-BSA is commonly composed of approximately 10 molecules of E2 attached to every BSA molecule. An E_2_-BSA solution equimolar in estradiol to a solution of estradiol alone would contain one-tenth the molarity of E_2_-BSA with respect to the concentration of estradiol alone. However the rate of E_2_-BSA binding is dependent upon the concentration of E_2_-BSA (rate = k [E_2_-BSA] [ER]). Even if every collision between E_2_-BSA and ER produced binding as successful as collisions between E2 alone and the ER, an E2-BSA solution equimolar in E2 would have approximately one-tenth the rate.

Taken together, these factors reduce the binding efficiency of E_2_-BSA to ER compared with free E_2_. However, once binding has occurred, the stability of the E_2 _molecule in the ER binding pocket may be only modestly impaired. This may explain how pre-incubation with E_2_-BSA results in successful binding, whereas immediate addition of E_2_-BSA does not have sufficient time to establish successfully bound forms. A similar rationale may explain our results and those of other investigators [15] that demonstrate specific cell surface binding of E_2_-BSA-FITC only to cells that express ERα. These studies typically employ at least a 30-minute incubation time with E_2_-BSA-FITC, which may be sufficient to result in significant binding. These factors strongly suggest that the rate of binding is an important consideration in experiments assessing potential interactions between E_2_-BSA and ER.

Although an estrogen receptor has not been directly isolated and characterized from the cell membrane, evidence other than E_2_-BSA activation of non-genomic effects has recently been reported that strongly supports the existence of a membrane ER. Immunocytochemistry using antibodies specific to several domains of the ERα stained only on the membrane of GH3 cells [26]. Membrane specific staining was prevented by treatment with antisense ERα mRNA or peptides that interfere with antibody binding. E_2 _conjugated to peroxidase also bound only to the membrane of pancreatic islet cells and this binding was competed by E_2 _[27]. The membrane impermeable E_2_-BSA-FITC was shown to stain only the membrane of ER deficient CHO cells transiently transfected with ERα and ERβ [15]. Moreover, ERα and ERβ interact directly with the membrane associated Src complex to trigger prostate cancer cell proliferation through the RAF-1/Erk-2 signal transduction pathway [5]. Lastly, we demonstrate that E_2_-BSA-FITC membrane staining is absent with ER deficient HeLa cells and present only on the membrane of cells that endogenously produce ER or HeLa cells that stably express mERα. Taken together these data strongly suggest that non-genomic effects of E_2 _are at least partially mediated by a membrane associated ER. However, whether the receptor is the classical nuclear ER translocated to the membrane or an ER unique to the membrane remains unanswered.

## Conclusions

The results presented here suggest that E_2_-BSA can bind to the estrogen receptor but the rate of binding is impeded due to steric and other considerations. Commercially available forms of the reagent are contaminated with dissociable E_2 _and should be purified prior to studies designed to demonstrate effects mediated through a membrane ER. Although we demonstrate that classical nuclear ERs can be translocated to the membrane, the conclusive identity of the endogenous membrane receptor awaits purification and sequencing of the putative membrane ER protein.

## Materials and Methods

### Establishment of ER stable transfectants

Full-length cDNA encoding the mouse ERα was cloned into a vector containing the CMV promoter driving the neomycin resistance gene (pcERα). HeLa cells maintained in MEM containing 10% fetal bovine serum under 5% CO2 were transfected with pcERα and successful transfectants (HeLa-ERα) were selected by survival in media containing the neomycin analog, G418 (400 ug/ml).

### Preparation of E_2_-BSA free of E_2_

400 ul of E_2_-BSA (10^-5 ^M in estrogen dissolved in 50 mM tris, pH 8.5, Sigma) was added to a centrifugal filter unit with a MW cut-off of 3,000 (Millipore) and centrifuged at 14,000 × g until 50 ul of retentate remained. The retentate was washed 3 times with 350 ul of buffer, recovered and volume adjusted to 400 ul.

### Binding of estradiol to purified estrogen receptor

^3^H-labeled E_2 _(NEN, specific activity 48 Ci/mmol, 10^-8 ^M) was incubated with recombinant ERα (.035 pM, Alexis Corp) for four hours at room temperature in binding buffer (10 mM tris, 10% glycerol, 2 mM DTT, and 1 mg/ml BSA). The binding of labeled E_2 _to ERα was competed by various concentrations of ultrafiltered E_2_-BSA or E_2 _(10^-9 ^to 3.5 × 10^-6 ^M in E_2_) added four hours prior to or concurrently with the addition of labeled E_2_. ERα was precipitated by the addition of a hydroxyapatite slurry (50% v/v in TE) and centrifugation at 10,000 × g. The pellet was washed three times with wash buffer (40 mM tris, 100 mM KCl, 1 mM EDTA, and 1 mM EGTA) and ^3^H-E_2 _binding determined by liquid scintillation counting.

### E_2_-BSA-FITC binding to cell membranes of ER producing cells

HeLa-ERα cells or mammary tumor cells (MC7, ATTC) were plated on glass cover slips and incubated with 500 ul of 10^-8 ^M (in estrogen) E_2_-BSA conjugated to FITC (E_2_-BSA-FITC, Sigma, 10 moles E2 and 3.5 moles FITC per mole BSA) or BSA-FITC (Sigma, equimolar to E_2_-BSA-FITC with respect to BSA) for 30 minutes at 4°C. Binding of E_2_-BSA-FITC to MC7 cells was competed by a 30 minute pre-incubation with E_2_, ICI-182,780, or E_2_-BSA (Sigma, 10^-7 ^to 10^-9 ^M). Cells were fixed and FITC staining visualized by confocal microscopy. Images were digitized, inverted to black on white, and pixel density for each cell determined by averaging the density across the cell membrane at four orthogonal points (Scion Image, Scion Corp).

The stability of E_2_-BSA-FITC during the incubation with MC7 cells was assessed by HPLC. E_2_-BSA-FITC (500 ul, 10^-8 ^M in estrogen) was added to empty wells and to wells containing MC7 cells prepared as above for 30 minutes at 4°C. 10 ul of supernatant was resolved using a C-18 reverse phase column (Xterra C-18 RP, 5 um, 4.6 mm × 250 mm, Waters). A multiple solvent deliver system (BIO CM 4000, Milton Roy) provided a methanol-water gradient from 80% methanol to 50% methanol over 30 minutes at a flow rate of one ml/minute. Peaks were detected by a scanning fluorescence detector (model 747, Waters) at an excitation wavelength of 495 nm and emission wavelength of 519 nm. Area under the curve was calculated using standard algorithms (Millenium Software). Assays were performed in triplicate.

### Estradiol binding studies

Subconfluent HeLa-ERα or native HeLa cells were trypsinized and aliquots (2 × 10^6 ^cells) incubated with several concentrations of ^3^H 17β-estradiol in the presence and absence of a 200-fold excess of cold 17β-estradiol for 30 min at 37°. Cells were then incubated on ice for 15 min, washed three times with 2 ml of ice cold 0.2% BSA-saline and pelleted by centrifugation at 1,5000 rpm for 10 min at 4°C. Cells were lysed by the addition of 100 ul of lysis buffer, vortexed and counted. Data was analyzed by Scatchard analysis.

## Competing interests

None declared.

## Authors' contributions

DB wrote the manuscript and performed binding assays. MK generated the stable cell lines. YT performed binding assays.
